# Food choice, embodied knowledge and circumscribed agency: factors influencing adolescent girls’ and boys’ dietary practices in three states in northern Nigeria

**DOI:** 10.1017/S1368980024001460

**Published:** 2024-10-10

**Authors:** Abigail Conrad, Akriti Singh, Sharmila Mysore, Stanley Nwosu, Michael Daniel Eveshoyan, Usman Ibraheem

**Affiliations:** 1 Results for Development, Washington, USA; 2 USAID Advancing Nutrition, Arlington, VA, USA; 3 Helen Keller International, New York, NY, USA; 4 John Snow, Inc., Arlington, VA, USA; 5 Helen Keller International, Abuja, Nigeria; 6 Independent Consultant, Nigeria

**Keywords:** Adolescent dietary practices, Nigeria, Diet quality, Communication platforms, Food choice

## Abstract

**Objective:**

The objective of this study was to explore adolescent dietary practices, related norms and acceptable communication platforms in northern Nigeria to inform future nutrition project design.

**Design:**

This was a qualitative formative research study. We used purposive sampling and conducted thirty focus group discussions with male and female adolescents aged 10–14 and 15–19 years (*n* 180) and six with adult influencers (*n* 36). We also administered a 24-h dietary recall with the adolescents using the Diet Quality Questionnaire.

**Setting:**

The study was conducted in urban and rural areas in three states in northern Nigeria.

**Results:**

Adolescents reported consuming six nutritious food groups the previous day on average. However, there was a wide disparity and only half consumed all five recommended food groups. Adolescents’ food choices were influenced by perceptions of the functional and physical benefits of nutritious foods and preferences for satisfying foods. Diverse foods were available in the food environment, but affordability constrained access to nutritious foods. Limited access to income and gender norms constrained adolescent agency over food choice. Girls, particularly those who were pregnant, had less agency related to food than boys. Adolescents thought that peers should be reached through group discussions, radio and phones, among other communication platforms.

**Conclusions:**

Adolescents consumed relatively diverse diets. Adolescent food choice was influenced by their embodied experience and knowledge related to nutrition and taste, home food environment and circumscribed agency. Opportunities exist to support healthy diets for adolescents by strengthening adolescents’ embodied knowledge, food environments and social support.

Adolescence is a period of rapid physical and emotional change when nutrition is critical for optimal growth and development. This is particularly relevant in Nigeria as 43 % of the country is under 14 years of age and 21 % is adolescent (10–19 years)^(1,2)^. In addition, 19 % of girls 15–19 years old nationally have given birth; this rate is much higher in the northern states of Bauchi (41 %), Kebbi (27 %) and Sokoto (32 %)^(3)^. Pregnancy during adolescence can have negative consequences for the mother and infant and perpetuate the intergenerational cycle of undernutrition^(4)^. However, many nutrition projects target adolescent girls who are already married, pregnant or mothers. Practitioners seldom design these projects for adolescents and instead adapt projects designed for older women. To address undernutrition, we must consider not only adolescent girls but also adolescent boys, as they grow up to be husbands, fathers and household decision-makers^(5)^.

Identifying interventions to support optimal adolescent nutrition practices requires examining data on nutrition status, dietary practices and effective communication platforms. Available studies indicate undernutrition is a serious concern in Nigeria and overweight/obesity is a rising issue for adolescents. Undernutrition is more common for younger adolescents (10–14 years) than older adolescents (15–19 years). An estimated 15 % of younger adolescent girls are underweight and 21 % are stunted^(3,6)^. Anaemia is also highly prevalent among adolescent girls – more than 40 % of girls 10–14 years and 53 % of girls 15–19 years are anaemic^(3,6)^. Although nationally representative data are unavailable for adolescent boys, a few studies conducted in the southern states reported that 3–25 % of boys 10–19 years old are underweight^(7–15)^.

While several studies have examined adolescent nutrition in Nigeria, little of this research has been done in the north^(16)^. Previous studies in the north have found that unequal intrahousehold food allocation, limited participation in decision-making and limited purchasing power negatively affect adolescent girls’ dietary intake^(5,17,18)^. In another study, adolescents reported household size and financial constraints limited animal-source food consumption^(17)^.

These studies vary in quality, adolescent sub-group (e.g. gender), demographic (mostly among in-school adolescents) and geographic coverage (mostly in southern states). They also rarely solicit input from adolescents on how to design projects that promote nutritious food consumption among their peers. Therefore, the objectives of this study were to explore the following to inform future nutrition project design: (1) dietary practices of adolescent boys and girls, (2) norms and aspirations that influence dietary practices and (3) acceptable communication platforms to promote healthy adolescent diets.

## Methods

### Study design

**Fig. 1:**
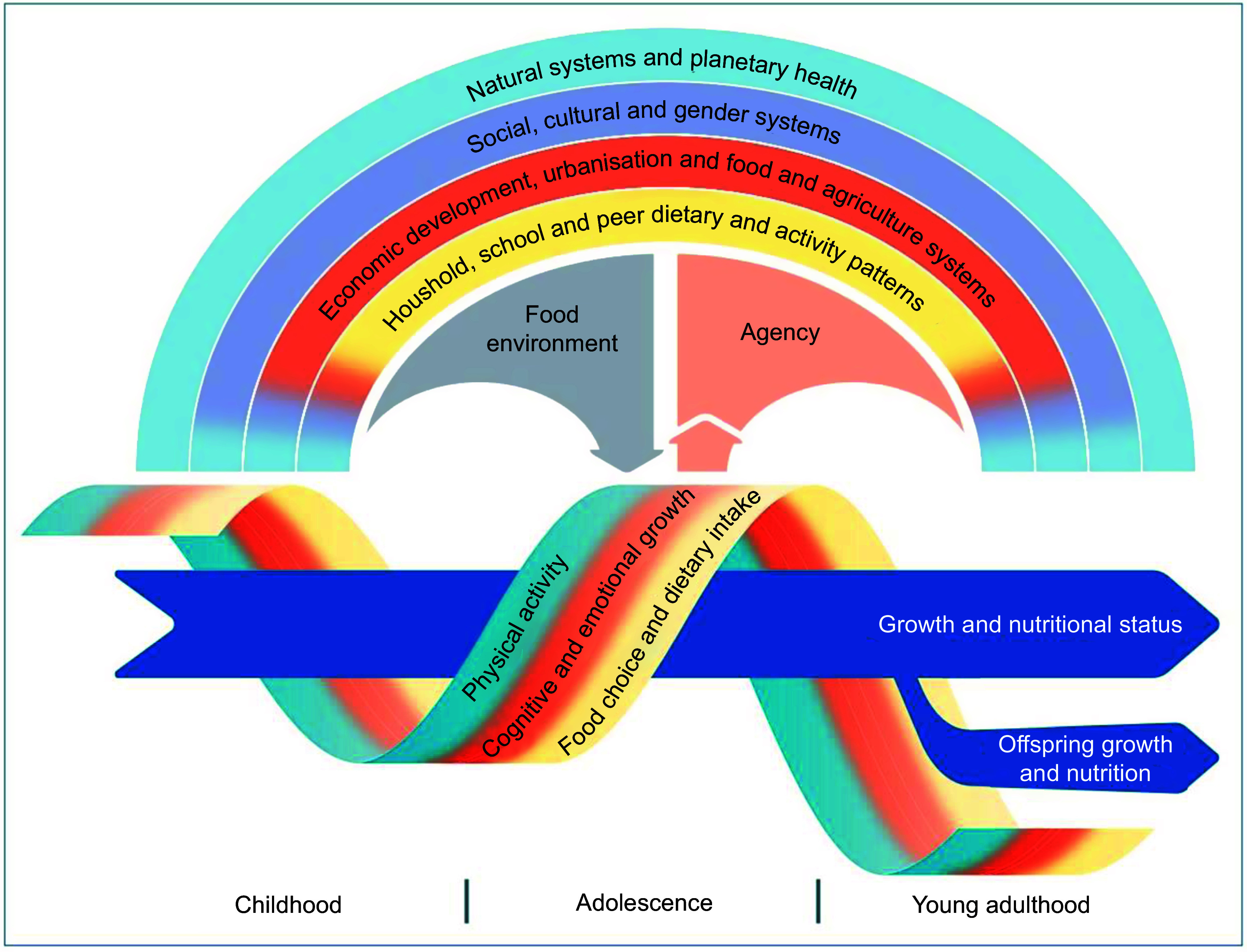
Conceptual model for factors influencing eating behavior of adolescents. A conceptual framework for adolescent growth and nutrition. Adolescence occupies a middle ground in human growth, affected by nutrition in earlier life but also setting trajectories for nutrition into later life and the next generation (blue arrows). The growth and maturation of physiological systems are affected by an adolescent’s immediate food environment (grey arrow), shaping dietary intake and norms, physical activity, and body image preferences (multicoloured ribbon). The food environment is shaped by cultural, economic, commercial and environmental ecosystems that extend down to the family and community settings in which adolescents are growing up. Conversely, adolescents have a growing agency (salmon arrow) to influence these ecosystems shaping their food environment

USAID Advancing Nutrition completed this formative research study using traditional and participatory qualitative research methods, a 24-h diet recall and secondary data. The topics in the guides were informed by Patton et al.’s conceptual framework for adolescent growth and nutrition (Fig. 1)^(19)^, although it was outside of the scope of this study to examine all factors in the framework. Following the conceptual framework, we present the findings for food choice and dietary intake as the key behaviours of interest, and then we present how those choices are influenced by the food environment (i.e. context in which consumers acquire, prepare and consume food^(20)^) and agency. The influence of social, cultural and gender systems and food and agriculture systems are integrated throughout the findings as cross-cutting factors in the conceptual framework.

We invited stakeholders to participate in workshops in each state to ensure local input in the study design, interpretation of the findings and development of nutrition project recommendations. Stakeholders included nutrition focal persons from relevant state ministries, parents of adolescents, community leaders, implementing partners and adolescent boys and girls.

### Study setting

We conducted this study in February 2023 in Bauchi, Kebbi and Sokoto States in northern Nigeria. Study sites were in six local government areas (LGA) in three livelihood zones: Sokoto millet, cowpeas, groundnuts and livestock; Sokoto-Rima-Kano riverine flood plain rice and fishing; and the central sorghum, maize, groundnut, cowpeas and sesame livelihood zones^(21)^. The rainy season typically extends from June to October and the lean season from July to September.

Sokoto had the LGA with the highest population density (20 863 people/kilometres (km)^2^) and Kebbi had the LGA with the lowest (78 people/km^2^). Based on a pilot study of a market-based food environment assessment (M-FEA) previously conducted by USAID Advancing Nutrition^(22)^, the selected communities in the LGA mostly had open-air markets and convenience stores. Some had small grocery stores, bread shops or bakeries, and street vendors. Many markets had vendors selling a variety of healthy foods and foods to limit year around, low quantities of advertisements and moderate produce quality. The M-FEA identified about 300 unique foods available, with the least diversity in Kebbi. The lowest cost of a healthy diet was $0·79–$3·27/d for an adult. Just prior to data collection for this study, food security was between minimal and stressed in Kebbi, minimal and crisis in Sokoto, and mostly minimal in Bauchi^(23)^.

### Study population and sampling

Within each state, we selected LGA included in the M-FEA to enable analysis of study data by key market food environment characteristics. This included one urban and one rural LGA in each state considering differences in demographics, food security status and agricultural characteristics. Within each LGA, we selected three communities based on proximity to key markets identified during the M-FEA. All selected communities were between 1 and 1·5 km from the markets. USAID Advancing Nutrition did not implement project activities in these communities other than the M-FEA and this study.

We conducted thirty-six focus group discussions (FGD), with 216 participants (*n* 180 adolescents, *n* 36 influencers). We conducted six FGD with each participant group in line with typical guidance^(24,25)^. We conducted a total of twelve FGD in each state: one FGD in a rural and one in an urban community for each adolescent group (pregnant girls 15–19 years old, non-pregnant girls 15–19 years old, younger adolescent girls 10–14 years old, older adolescent boys 15–19 years old and younger adolescent boys 10–14 years old) and with influencers. We included the following types of influencers: community leaders, religious leaders, teachers, elders, parents of adolescents and community health workers or volunteers.

The LGA provided introductions to community leaders, who assisted in identifying diverse adolescent FGD participants (e.g. socio-economic status, in-school and out-of-school, married and unmarried) and influencers. We asked or ascertained in a culturally appropriate manner if older adolescent girls were pregnant before recruiting them. We purposively selected six participants per FGD to ensure moderators could solicit input from all participants and support adolescents in activities, particularly those with limited literacy^(26)^.

### Data collection techniques and procedures

#### Focus group discussions

We used FGD for this exploratory study as an appropriate way to gather data on common experiences and perceptions^(26)^, to engage adolescents for data collection and to accommodate the short study timeline. We conducted separate FGD for each adolescent group and male and female influencers. FGD were completed in Hausa and audio-recorded with consent. FGD with adolescents covered topics including adolescent agency related to food, food security, trusted information sources on nutrition and suggestions for interventions to encourage peer consumption of nutritious foods. The FGD included participatory exercises such as creating daily activity calendars, ranking exercises and drawing^(27)^. FGD with influencers explored factors that influence adolescents’ diets, the role of family and community members in adolescent diets, food security and suggestions for interventions.

#### Diet Quality Questionnaire with adolescents

Using the Diet Quality Questionnaire (DQQ) developed for Nigeria^(28)^, data collectors administered a 24-h diet recall to the adolescent FGD sample (*n* 180). The dietary recall was completed individually with each adolescent after the FGD. The DQQ asked about consumption of twenty-nine food groups the adolescent ate inside or outside the home. This list-based dietary recall method uses sentinel food lists^(29)^.

### Data analysis

Translators transcribed the Hausa FGD recordings into English. The study team then organised and coded them using ATLAS.ti software^(30)^. We used predetermined codes based on the research questions as well as codes that emerged from the data. Four team members coded 10 % of the transcripts to establish intercoder reliability and independently coded the remaining transcripts. During this initial coding, the team reflected on how our personal experiences and biases influence interpretation and the importance of avoiding making assumptions and judgements when coding. One team member reviewed all coded transcripts for consistency and quality. We used secondary data from the M-FEA on community distance to market and key market food environment characteristics (e.g. cost of diet and food availability) to explore patterns in the qualitative data by market food environment characteristics. We also explored variations in patterns by urban/rural, influencers and adolescent categories.

We analysed data on twenty-nine food groups from DQQ in R 4.2.3 software^(31)^. The indicators of diet quality examined were food group diversity score, minimum dietary diversity for women of reproductive age (MDD-W), consumption of all five recommended food groups (all-five), non-communicable disease (NCD)-protect score, NCD-risk score and global dietary recommendations (GDR) score^(28)^.

## Results

### Participant description

As planned, there were thirty-six participants per category. The adolescents’ average household size was ten people (Table 1). None of the adolescent boys were married, but most of the older pregnant girls and some older non-pregnant girls were married. Half of adolescents attended conventional school during the day and Islamic school in the evening, while the other half only attended Islamic school during the day or evening. A lower proportion of pregnant girls 15–19 years old attended school compared with other adolescent groups. The mean age of the influencers was 40 years, their average household size was 11 and almost all had an adolescent in their household.

**Table 1:** Background characteristics of FGD participants (adolescents and influencers)

	All adolescents		Girls (10–14 years)		Non-pregnant girls (15–19 years)		Pregnant girls (15–19 years)		Boys (10–14 years)		Boys (15–19 years)		Influencers	
	*n*	%	*n*	%	*n*	%	*n*	%	*n*	%	*n*	%	*n*	%
*n*	180		36		36		36		36		36		36	
Age														
Mean	15·66		13·39		16·92		18·14		12·42		17·42		40·22	
sd	2·58		0·77		1·38		0·93		1·40		1·23		10·89	
Gender (*n*, %)														
Female	108	60 %	36	100 %	36	100 %	36	100 %	0	0 %	0	0 %	18	50 %
Household size														
Mean	9·68		12·03		11·25		5·75		9·06		10·31		11·08	
sd	5·78		8·07		5·59		3·81		3·42		4·84		5·97	
School attendance (*n*, %)	90	50 %	21	58 %	15	42 %	4	11 %	24	67 %	26	72 %		
Islamic school attendance (*n*, %)	148	82 %	36	100 %	31	86 %	22	61 %	29	81 %	30	83 %		
Married, (*n*, %)	39	27 %	0	0 %	7	19 %	32	89 %	0	0 %	0	0 %		
State (*n*, %)														
Bauchi	60	33 %	12	33 %	12	33 %	12	33 %	12	33 %	12	33 %	12	33 %
Kebbi	60	33 %	12	33 %	12	33 %	12	33 %	12	33 %	12	33 %	12	33 %
Sokoto	60	33 %	12	33 %	12	33 %	12	33 %	12	33 %	12	33 %	12	33 %
Residence														
Urban	90	50 %	18	50 %	18	50 %	18	50 %	18	50 %	18	50 %	18	50 %
Rural	90	50 %	18	50 %	18	50 %	18	50 %	18	50 %	18	50 %	18	50 %
Position														
Parent													13	36 %
Teacher													10	28 %
Health worker													5	14 %
Other^*^													8	22 %
Adolescents living in the household (*n* (%))													30	83 %

FGD, focus group discussion.

* Other included community leaders and elders, such as traditional birth attendants.

### Dietary intake

**Fig. 2:**
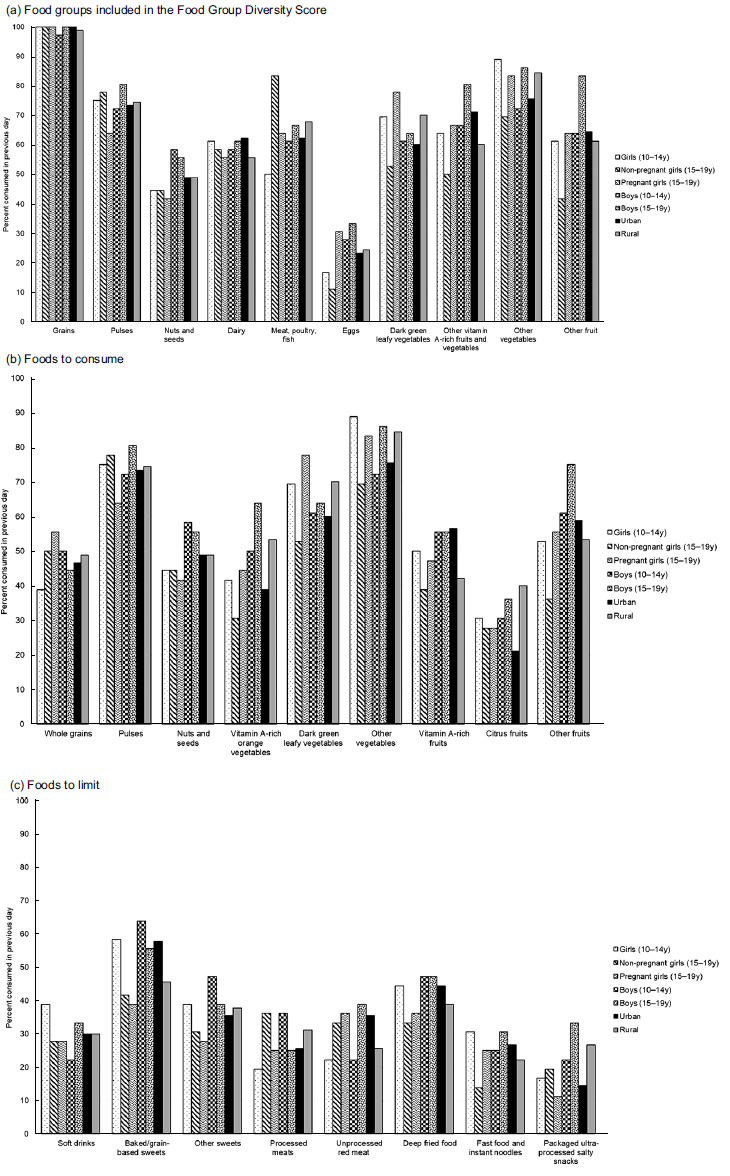
Distribution of different food groups consumed by adolescent boys and girls. (a) Food groups included in the food group diversity score, (b) foods to consume and (c) foods to limit

Data from the DQQ showed that on average, adolescents reported consuming six out of ten food groups the previous day (Table 2). However, there was a wide disparity, with some adolescents reporting consumption of only one food group and others reporting consumption of all ten. Approximately a quarter of girls 15–19 years old did not meet the MDD-W, with one reporting that she consumed only one food group the previous day. Only half of adolescents (56 %) consumed all five recommended food groups. The recommended food groups missing in the diet of the other half of the adolescents were whole grains (48 % consumed), nuts and seeds (49 %) and eggs (24 %). One notable difference was that more adolescent girls 15–19 years old consumed meat, fish and poultry (83 % *v*. 50–67 %), but fewer dark green leafy vegetables (53 % *v*. 61–77 %) than other adolescent categories (Fig. 2; online Supplementary Table 1). In the previous day, approximately a third or more adolescents consumed several food groups to limit such as baked/grain-based sweets (52 %), deep fried foods (42 %), other sweets (37 %), unprocessed red meat (31 %) and soft drinks (30 %), particularly in urban areas.

**Table 2:** Diet quality of adolescent boys and girls

	All		Girls (10–14 years)		Non-pregnant girls (15–19 years)		Pregnant girls (15–19 year)		Boys (10–14 years)		Boys (15–19 years)		Urban		Rural	
	Mean	Range	Mean	Range	Mean	Range	Mean	Range	Mean	Range	Mean	Range	Mean	Range	Mean	Range
*n*	180		36		36		36		36		36		90		90	
FGD score^*^ (0–10)	6·43	1–10	6·31	2–10	5·89	2–10	6·47	1–10	6·39	1–10	7·11	1–10	6·41	1–10	6·46	1–10
MDD-W (≥ 5)																
* n*	53				26		27									
%	74 %				72 %		75 %									
GDR^†^ score (0–18)	11·26	6–17	11·31	8–16	10·69	6–15	11·69	7–15	11·14	8–15	11·47	8–15	11·13	6–17	11·39	7–16
NCD – protect score (0–9)^‡^	4·98	0–9	4·92–	1–9	4·28	0–9	4·97	1–9	5·11	0–9	5·61	0–9	4·80	0–9	5·16	1–9
NCD – risk score (0–9)^§^	2·93	0–9	2·89	0–9	2·72	0–7	2·53	0–8	3·22	0–9	3·28	0–9	2·67	0–9	2·77	0–9
All five recommended food groups^||^																
* n*	100		19		17		19		21		24		50		50	
%	56 %		53 %		47 %		53 %		58 %		67 %		56 %		56 %	

FGD, focus group discussion; MDD-W, minimum dietary diversity for women of reproductive age; GDR, global dietary recommendations; NCD, non-communicable disease.

* Food group diversity score includes ten food groups: (1) grains, white roots and tuber, and plantains; (2) pulses (beans, peas and lentils); (3) nuts and seeds; (4) dairy; (5) meat, poultry and fish; (6) eggs; (7) dark green leafy vegetables; (8) other vitamin A-rich fruits and vegetables; (9) other vegetables; (10) other fruits.

† GDR score = NCD – Protect – NCD – Risk + 9; measures adherence to global dietary recommendations protective against non-communicable diseases.

‡ NCD – protect score measures adherence to global dietary recommendations on foods to consume: (1) whole grains; (2) pulses; (3) nuts and seeds; (4) vitamin A-rich orange vegetables; (5) dark green leafy vegetables; (6) other vegetables; (7) vitamin A-rich fruits; (8) citrus; (9) other fruits.

§ NCD – risk score measures adherence to global dietary recommendations on foods to limit including: (1) soft drinks; (2) baked/grain-based sweets; (3) other sweets; (4) processed meats; (5) unprocessed meat; (6) deep fried food; (7) fast food and instant noodles; (8) packaged ultra-processed salty snacks.

|| All five recommended food groups include (1) starchy staples, (2) vegetables, (3) fruits, (4) pulses, nuts, and seeds and (5) animal-source foods.

During the FGD, common dishes adolescents reported consuming during the previous day for meals were swallow (starchy, stiff dough) made from maize, millet, guineamaize, rice or sorghum with soup of okra or baobab leaves; rice with stew/beans/pepper and oil; and pap (white corn meal) with kosai (bean cake). Several adolescents (more in urban areas) also reported eating pasta and instant noodles. A few adolescents reported eating milk, eggs, chicken and fish, but overall, few discussed consuming animal-source foods. This may be because meat was included in small quantities in the stew or soup they consumed. Common snacks adolescents reported consuming were fruits (orange, mango and banana), awara (tofu) and kosai. More adolescents in urban than rural areas reported snacking except for in Kebbi State (online Supplementary Table 2).

### Food choice

#### Perceptions of nutritious and non-nutritious foods

Adolescents perceived a range of foods to be nutritious and associated different functional and physical effects to these foods (Table 3). Among the foods they ate the previous day, they commonly reported that beans and water are nutritious, in addition to rice, soup, swallow made with maize or rice, yam, soya bean and milk. In contrast, several influencers noted that fruits, vegetables (especially leafy green vegetables), beans, soya bean and animal-source foods are nutritious.

**Table 3:** Factors and perceptions influencing adolescent food choice

Themes	Summary	Illustrative quote
Perceptions of nutritious foods	● Food that are filling	Moderator (M): ‘Why do you say tuwo [swallow] is nutritious?’ Participant (P): ‘Because it gives satisfaction.’ P: ‘It is filling.’ P: ‘It is always available, we grew up eating it.’ (Bauchi, rural, pregnant girl 15–19 years)
	● Functional benefits such as strength to do work, aid in growth, contribute to intelligence, ‘give blood to the body’, satisfy hunger and lead to good health ● Physical benefits such as making them fat (desirable), look fresh, have glowing skin and no wrinkles	P: ‘If I eat [rice meal with stew] it increases my health and it increases blood.’ M: ‘What does blood do to your health?’ P: ‘You won’t be hungry and you will be strong.’ (Kebbi, urban, boy 10–14 years) ‘When you start eating nutritious foods, your body and skin will look different: fat, shiny and healthy, and fair in complexion.’ (Bauchi, rural, girl 10–14 years)
Perceptions of non-nutritious foods	● Food that are not filling	M: ‘Why do you say carrots and tomatoes are not nutritious?’ P: ‘because it doesn’t give satisfaction.’ (Bauchi, rural, boy 15–19 years)
	● Makes them thin, ill and weak	P: ‘He will always be sick and lose weight.’ (Sokoto, rural, boy 10–14 years)
Perceptions related to girls’ reproductive health	● Girls considered sweets non-nutritious during menstruation because they were advised to avoid sweets	P: ‘Yes, we are advised to stay off anything with sugar when we are menstruating to avoid menstrual pains.’ (Sokoto, rural, girl 10–14 years)
	● Girls were advised to eat nutritious foods, mainly by healthcare providers	P: ‘Yes, like beans, lettuce, spinach that increases the health of the body.’ (Kebbi, urban, girls 15–19 years)
	● Only some older pregnant adolescent girls knew about iron folic acid supplements.	M: ‘Do you all know what iron and folic acid are?’ P: ‘No, we don’t know.’ (Sokoto, rural, pregnant girl 15–19 years)
Food preferences		
Preferred foods	● Adolescents primarily preferred staples, beans, and vegetables ● Rice was the most common favourite food	● ‘I feel satisfied after eating rice… It gives me energy to enable me to help my mother with house chores like sweeping and washing.’ (Sokoto, rural, adolescent girl 15–19 years) ● ‘I choose spaghetti and the reason I love it is because early in the morning before I start my day I do buy it for breakfast to eat; so I am used to it that’s why I love it.’ (Bauchi, urban, adolescent boy 15–19 years)

Adolescents described how nutritious foods provide strength to do work, aid in growth, contribute to intelligence, increase blood, satisfy hunger and lead to good health. Physical benefits they described were outward signs of health, including that nutritious foods make a person fat (which was desirable), have glowing skin and no wrinkles. Some also noted that one has to eat to one’s satisfaction (fullness) to benefit from nutritious foods.

There was some variation in nutritious food perceptions between boys and girls. More older boys than younger boys mentioned that nutritious foods make one’s skin glow and that this makes them attractive to girls. Girls talked about the value of nutritious foods during menstruation and childbirth. Across age groups, girls explained that nutritious foods make their skin glow, which some older girls noted helps attract future husbands.

Adolescents provided less consistent responses about non-nutritious foods than nutritious ones, some perceptions about nutritious and non-nutritious foods were contradictory, and they held some misconceptions about non-nutritious foods. Several adolescents mentioned rice as a non-nutritious food, while others perceived liquids such as tea, water and milk to be non-nutritious. Others considered awara, vegetables (e.g. carrots, tomatoes and cucumbers), fruits (e.g. oranges and mango) and staples such as rice, fura (dough balls made from millet) with nono (fermented milk drink), cassava and pasta as non-nutritious. Some adolescents noted that foods that they do not eat often are non-nutritious.

As with nutritious foods, adolescents largely identified non-nutritious foods by their physical effects. Adolescents reported that non-nutritious foods provide no satisfaction, leaving them hungry. They said they only consume these foods for the taste or that they did not see any change in their bodies after eating these foods. When asked about the negative consequences, adolescents shared that non-nutritious foods make them slim, ill and weak. Except for younger adolescent boys, all adolescent participant groups mentioned that not eating nutritious foods would result in dull or dark skin. Boys specifically mentioned that a lack of nutritious food would result in stunted growth, while girls mentioned that lack of good nutrition would result in loss of blood. A few older adolescent girls mentioned that some foods such as chinchin (crunchy fried snack) and bread made the body heavy (considered a negative), but adolescents largely did not talk about being overweight in a negative light.

#### Food perceptions related to girls’ reproductive health

Adolescent girls shared nutrition perceptions related to their reproductive health which influenced their food choice during menstruation and pregnancy (Table 3). Girls typically said that during menstruation, they are advised to avoid sweet foods, cold water and cold food and to consume nutritious foods to replenish blood.

Pregnant girls said that healthcare providers advised them to eat foods like beans, leafy green vegetables, animal-source foods and awara. Despite receiving dietary advice from health workers, only a few pregnant adolescent girls living in urban areas discussed taking iron folic acid supplements in FGD.

#### Food preferences

Food preferences were a key driver of adolescent food choice. They described preferring a range of foods, but primarily preferred staples, beans and vegetables. Taste was the most important characteristic of preferred foods, with adolescents saying they tasted good, were delicious or ‘loved’ the food. Sometimes, they associated their preferences with how the food affects their body, such as giving energy, building the body or giving strength (particularly among boys). Rice, such as with beans or a vegetable soup, was the most preferred food across adolescent groups. They shared that they enjoy rice, and it is delicious, satisfying, and sweet, keeps you full, increases strength, gives energy and increases blood in the body. A few said they like rice because they eat it often and are used to it, while others said they like it because they do not always get to eat it. In urban areas and markets with a high number of advertisements (based on the M-FEA), adolescents expressed an equal preference for rice and spaghetti or other noodles like Indomie (instant noodles). Other common foods adolescents liked were beans, soup (e.g. sorrel, Baobab leaves and okra), swallow made from maize, guinea corn (sorghum), or rice, and stew. Animal-source foods and fruits were not common favourites.

#### Aspirations

Peer aspirations can influence adolescent food choice as they consider the perceived status and benefits of foods. Adolescents described functional benefits of foods that support their aspirations, like giving strength to do work for boys and to help have a healthy pregnancy and childbirth for girls. Adolescent girls expressed aspirations to get married, have children and a safe delivery, and be good mothers. Married adolescent girls aspired to be good wives and to support their husbands’ businesses, so their families could thrive. Adolescent boys often aspired to become influential people, such as religious leaders, to protect their community. Girls aspired to get a full education to help other women in their community, while boys aspired to gain an education to better their businesses.

### Food environment

#### Household food access

Adolescents primarily ate food at home, making the home central to adolescents’ food environment (Table 4). Influencers reported accessing a wide variety of foods across seasons from their own production and market purchases. Influencers in rural and urban areas maintained farms, in which they grew crops such as millet, guinea corn, beans and rice.

**Table 4:** Food environment drivers of adolescent diets

Factor	Summary	Illustrative quote
Household food access	● Range of foods were available from household production and accessible to purchase at the market or farmgate	● ‘Beans are affordable. Sometimes we get fish either through buying or fishing ourselves depending on the season. Even if someone isn’t rich, we can afford things like millet.’ (Kebbi, urban, male influencer) ● ‘We are rural people, what we use mostly here is millet, guinea corn… We also farm vegetables which we also include in our food.’ (Bauchi, rural, male influencer)
	● Influencers reported constraints affording enough nutritious foods for household	● ‘Again money matters because everyone has to help his family and it is barely enough.’ (Sokoto, urban, male influencer) ● ‘They do get nutritional food but it’s usually not enough.’ (Sokoto, rural female influencer)
Adolescent food access	● Adolescents primarily ate the same food as other household members ● Parents feel they have the responsibility to provide nutritious food for unmarried adolescent children ● Husbands have the responsibility to provide nutritious foods for married adolescent girls	● ‘A father should just try his best to provide [his daughter] with all the nutritious food she needs that will build her body and increase her energy.’ (Kebbi, urban, male influencer) ● ‘The mother or sister [cook]… Because they are women and when the father provides, they have to prepare it because it is their duty.’ (Kebbi, urban, adolescent boy 10–14 years) ● ‘We only eat what our husband brings to the table.’ (Sokoto, rural, pregnant adolescent girl 15–19 years)

Influencers and adolescents reported ready access to markets, and all lived in communities within a 40-min walk of a market (based on the M-FEA). Influencers said they regularly purchased food in markets and at the farmgate, particularly foods they did not produce themselves. While participants and the M-FEA showed availability of diverse foods in markets, influencers reported lacking enough money to purchase the desired quantities of these foods for their families, including enough nutritious foods for adolescents. Influencers and participants in rural areas, Sokoto and communities with a high cost of diet in nearby markets (based on the M-FEA) discussed food access constraints more than other participants.

#### Adolescent food access

Adolescents primarily ate the same food as others in their household and were thus mostly dependent on household food access. Influencers and adolescents characterised provision of sufficient, nutritious for adolescents as a strong familial responsibility, with roles prescribed by sociocultural norms. For unmarried adolescents, fathers were responsible for earning income to purchase food for the family and leaving the house to find food. For married adolescent girls, husbands were largely responsible for providing food.

### Agency

#### Adolescent food acquisition and food choice

Within the context of adolescents’ food environments, they exerted degrees of agency or self-described ‘freedom’ in acquiring food and making food choices depending on gender, age and marital status (Table 5). Adolescents sometimes reported eating food that other family members did not. These were typically snacks that they ate if they were outside of the home, alone at home or if there was not enough of a snack to share. Adolescents reported less strict norms around sharing and serving snacks than meals because they said fathers do not eat snacks, are not always home when they were eaten and do not think snacks are satisfying due to their small quantity.

**Table 5:** Adolescent food-related agency

Factor	Summary	Illustrative quote
Adolescent food acquisition and food choice	● Adolescents could eat food alone outside of the home, when alone, or if there was not enough of a snack to share ● Adolescents have more agency to choose snacks than meals ● Adolescents were dependent on having access to money if they wanted to buy snacks for themselves. Girls had less agency to do this. ● Pregnant girls, most of whom were married, had less agency over food consumption than other adolescent groups.	● ‘Only if you take it home and they see it, it will be shared but if you buy it outside you can eat without sharing.’ (Kebbi, urban, adolescent girl 10–14 years) ● ‘We usually buy [snacks] ourselves, with our money.’ (Sokoto, rural, adolescent boy 15–19 years) ● ‘Yes there is [a difference] because an adolescent girl is always with you. But a boy is sometimes trying to be on his own so you worry less about him, he can go out and eat outside with his money; but a girl child cannot do that, she can only eat what is provided at home.’ (Kebbi, urban, male influencer) ● ‘The unmarried has less responsibility than the married one and she is free to do whatever she wants to do, unlike the married one whatever she does is with permission.’ (Bauchi, urban, pregnant girl 15–19 years)
Adolescent influence on household food choice	● Girls had some influence on household cooking decisions ● Married, older adolescent girls made cooking decisions ● Some older boys used their own money to purchase food for the household	● ‘We do involve [girls] by asking them what they think we should cook today. Or to go and ask their father what we should cook.’ (Bauchi, urban, female influencers) ● ‘The wives are responsible for preparing the food we eat at home.’ (Sokoto, rural, pregnant adolescent girl 15–19 years) ● ‘The boys are involved because sometimes they support the shopping with their money. On a particular day that he makes more money, he will buy foods like yam, spaghetti for the family.’ (Bauchi, rural, male influencer)
Intrahousehold food allocation	● Mothers served food, with adolescents served after fathers and young children. ● Married girls served food in their households, and served themselves last (or along with co-wives).	● ‘You put the children’s food separately and put yours with your fellow women (co-wives).’ (Bauchi, urban, pregnant adolescent girl 15–19 years)

Adolescents across groups and geographies were able to buy food with money they earned; however, older adolescent boys had greater access to income and mobility to do this than other adolescent groups. Adolescents typically purchased snacks, which were often nutritious, like kosai and awara. Occasionally, older adolescent boys purchased meals outside of the home if they did not like what their mother made for dinner.

Girls had less mobility to leave home than boys. Younger girls, who were all unmarried, talked more about purchasing snacks for themselves while they were at the market or hawking (selling goods on the street) than older girls did. Pregnant girls expressed the least agency over food than other adolescent groups as few reported purchasing or eating food that other family members did not. As most pregnant girls were married, they described being reliant on their husband to make food purchases, having more responsibility and less freedom than unmarried girls. They may also have had limited mobility while pregnant.

#### Adolescents’ influence on household food choice

Married girls made cooking decisions for their household, while unmarried adolescents typically had limited influence over household food choices despite heavy involvement in food preparation and cooking for girls and farming for boys. Some female influencers said they solicited unmarried girls’ opinions on what to cook and a few noted they take adolescent’s food preferences into account when deciding what to cook. Other influencers said only parents decide what to cook. Some older boys had a larger influence on household food choices because they used their own money to purchase food for the household.

#### Intrahousehold food allocation

Mothers mediated food access for unmarried adolescents by determining serving order and the quantity of food distributed. Adolescents were served food after their father and young children in the household, and boys and girls usually ate separately. Married adolescent girls served themselves food last as the mother and wife (or along with co-wives).

### Activity patterns and communication platforms

To inform the design of interventions to reach adolescents, we explored daily activity patterns and communication platforms that they thought projects could use to engage them and their peers. Adolescents described active daily schedules, which typically included frequenting school, markets, farms and gathering with friends (see online Supplementary Table 3). Adolescents discussed a wide range of platforms to engage peers and were motivated to share nutrition information with their peers. Some thought group discussions would be valuable. They also thought radio, phones and social media (WhatsApp and Facebook), and inviting healthcare workers to discuss nutritious foods and healthy eating in schools would reach adolescents. Adolescents reported that the most trusted sources of information were parents, phones and social media, radio and television, health workers and nutrition experts, and teachers.

## Discussion

This study used qualitative methods, a 24-h dietary recall and secondary data to explore the dietary practices of adolescents (10–19 years) in three states in northern Nigeria. We found that adolescent diets were diverse, with an average of six food groups consumed the previous day, but only one in five adolescents ate all five recommended food groups the previous day. About a third of adolescents also consumed foods to limit, such as baked/grain-based sweets, deep-fried foods, unprocessed red meat and soft drinks. While dietary diversity for adolescents has not been estimated at the national level in Nigeria, our study found higher dietary diversity among adolescents than other studies in the north^(18)^ and south^(32)^ and than adults in a study that administered the DQQ at the national level^(28)^. But similar to our study, the national DQQ survey found a large proportion of adults consumed vegetables and animal-source foods, food groups likely to be missing from diets were whole grains and nuts and seeds, and about a third of the population consumed foods to limit^(28)^. It is important to note that our study sample was not representative and our results may be higher because the study was conducted right after the harvest season and in communities close to markets^(33)^.

Our findings confirm the importance of and interplay between factors influencing adolescent food choice and dietary intake in Patton et al.’s conceptual framework^(19)^. Different forms of embodied experience and knowledge were key drivers of adolescent food choice. Rather than providing biomedical explanations about nutrition, adolescents primarily discerned and came to understand the nutritional qualities of foods based on how they make them feel when they eat them and the effects (or lack thereof) they experienced. Adolescents perceived nutritious foods to be those that are filling and that have a positive effect on the body, thus expressing embodied nutrition knowledge based on bodily experience^(34)^. Despite adolescents’ reports that health workers were trusted sources of information, they did not readily discuss receiving dietary recommendations from the health sector, apart from some pregnant girls who received dietary advice and iron folic acid supplements through health clinics.

We found that taste was a key driver of adolescent food choice, in line with a global study on adolescent food practices that included Nigeria^(35)^. Adolescents focused on the taste of foods when describing their food preferences, such as when describing favourite dishes of rice with beans or a vegetable soup as delicious and sweet. They also described a positive bodily response to food, such as keeping you full and giving energy, as part of what makes a food desirable. Adolescents perceived these positive bodily responses as supporting their life aspirations. Adolescents also discussed preferring foods they have access to. Adolescents living in urban areas and near markets with a high number of advertisements preferred spaghetti and other noodles more than other adolescents, suggesting an interplay between taste and exposure to foods. Such an association between food advertising and adolescent food preferences has been found in high-income countries^(36–38)^. Adolescents’ tastes were developed within and shaped by their food environment, as taste is not simply a sensory experience but is structured and shaped by food access, socio-economic conditions, power and social boundaries^(39–41)^.

As other studies have shown, adolescent food choice was also influenced by the food environment^(36)^. The home food environment was the most influential sphere of their food environment as they primarily had access to and ate food provided by their parents (or husbands for older married adolescent girls) within the home. This may vary by context within Nigeria as another study in Nigeria found that adolescents often ate alone^(35)^. Parents (and husbands for married girls) had strong familial responsibility to provide nutritious foods for adolescents. Diverse foods were available in study food environments, but families were not always able to afford sufficient, nutritious foods for adolescents.

Adolescents possessed and exercised agency related to food as has been found in other contexts^(36,42)^, but this agency was significantly structured and circumscribed by age, gender and marital status for girls as has been found by other studies in Nigeria. Adolescents were largely not involved in decision-making around food acquisition, purchase and preparation, except for married adolescent girls. Adolescents had some agency to make food purchases and consume food on their own, particularly snacks, with older boys having the most agency and pregnant girls having the least. Similar to our findings, in Sokoto State, married adolescent girls 15–19 years old noted that their husbands made decisions about what food to purchase and prepare, but wives were responsible for deciding who ate first^(5)^. We also found that older boys were more likely to purchase foods for personal consumption from the market than girls, likely due to norms around control of income. A study conducted in the northern states of Kano, Katsina and Jigawa found that only 40 % of girls had full control over their incomes, while others had partial or no control with older male siblings or fathers exercising control over the money the girls earned^(43)^. A study conducted among adolescent girls 10–19 years old in urban Borno State reported that more than half of the participants said it was hard to buy, cook and store nutritious foods, primarily due to their current food budget^(18)^. At mealtimes, we found that adolescents were served after fathers and young children and married adolescent girls served themselves last. In Bauchi State, adolescent girls reported receiving smaller food portions compared with their adolescent male siblings^(17)^. This gendered food agency has been found among adolescents in other countries in food environments classified as traditional^(36)^.

This study aimed to help fill significant evidence gaps about adolescent dietary practices in northern Nigeria to inform future USAID investments in the region. Our findings confirm those from other contexts that nutrition projects need to be tailored for adolescents and account for local sociocultural and gender norms. Our findings also confirm the need to address the multifaceted factors that influence adolescent diets, including to strengthen food environments and reinforce social and family support for healthy adolescent diets^(36,44,45)^. Interventions should value and enhance adolescents’ embodied nutrition knowledge, engage adolescents as change agents and bolster adolescent agency^(46)^. Our findings also point to pregnant adolescent girls 15–19 years as a particularly vulnerable adolescent group in northern Nigeria that projects should engage.

The relatively small number of sites limited this study, and it may not reflect the diversity in the three states, particularly experiences of communities living in remote or insecure areas. There may have been response bias related to social desirability or to garner project support. In addition, we collected limited demographic information about participants and did not measure food security. Future studies should conduct dietary recalls with a representative sample of adolescents, disaggregate data for married and unmarried older adolescent girls, include husbands of adolescent girls in data collection and use participant observation to further explore dietary practices and sociocultural and gender norms.

### Conclusion

Our findings contribute to the limited evidence base on adolescent dietary practices and their drivers in northern Nigeria and in low- and middle-income countries more broadly. We explored the interplay between and the complexity of factors in Patton et al.’s framework on adolescent eating behaviour. We found that adolescents’ embodied experience and knowledge – related to nutrition and taste – produced key ways of knowing that shaped their food choice. Their home food environment and sociocultural and gender norms shaped and circumscribed their agency to choose what and how to eat. We recommend further research on adolescent dietary practice in northern Nigeria and use of these findings to inform future project design, including by strengthening adolescents’ embodied knowledge, food environment and social support for healthy diets.

## Supporting information

Conrad et al. supplementary material Conrad et al. supplementary material

## Supplementary material

For supplementary material/s referred to in this article, please visit https://doi.org/10.1017/S1368980024001460

## References

[1] UN Population Fund (2022) World Population Dashboard Nigeria. https://www.unfpa.org/data/NG (accessed 13 July 2023).

[2] Samuel F (2021) Securing the future of Nigerian adolescents through nutrition: a position paper of the Nutrition Society of Nigeria. Niger J Nutr Sci 42, 1–8.

[3] Nigerian Population Commission & ICF (2019) Nigeria Demographic and Health Survey 2018. Abuja, Nigeria; Rockville, MD: Nigerian Population Commission and ICF.

[4] Norris SA , Frongillo EA , Black MM et al. (2022) Nutrition in adolescent growth and development. Lancet 399, 172–184. 34856190 10.1016/S0140-6736(21)01590-7

[5] International Medical Corps (2015) Case Study on Adolescent Inclusion in the Care Group Approach—the Nigeria Experience. Washington, DC: International Medical Corps.

[6] Federal Government of Nigeria & International Institute of Tropical Agriculture (2022) National Food Consumption and Micronutrient Survey 2021: Preliminary Report. Ibadan: International Institute of Tropical Agriculture.

[7] Adeomi AA , Adelusi IO , Adedeji PO et al. (2019) Nutritional status and cardiometabolic health among adolescents; findings from southwestern Nigeria. BMC Nutr 5, 45. 32153958 10.1186/s40795-019-0308-5 PMC7050742

[8] Eme PE , Onuoha NO & Mbah OB (2016) Fat-related anthropometric variables and regional patterns of body size and adiposity of adolescents in Aba South LGA, Abia State, Nigeria. Food Nutr Bull 37, 401–408. 27147246 10.1177/0379572116645917

[9] Mustapha RA & Sanusi RA (2013) Overweight and obesity among in-school adolescents in Ondo State, Southwest Nigeria. Afr J Biomed Res 16, 205–210.

[10] Odunaiya NA , Louw QA & Grimmer KA (2015) Are lifestyle cardiovascular disease risk factors associated with pre-hypertension in 15–18 years rural Nigerian youth? A cross sectional study. BMC Cardiovasc Disord 15, 144. 26537355 10.1186/s12872-015-0134-x PMC4632346

[11] Omobuwa O , Alebiosu C , Olajide F et al. (2014) Assessment of nutritional status of in-school adolescents in Ibadan, Nigeria. S Afr Fam Pract 56, 246–250.

[12] Ubesie A , Okoli C , Uwaezuoke S et al. (2016) Affluence and adolescent obesity in a city in south-east Nigerian: a cross-sectional survey. Ann Trop Med Public Health 9, 251.

[13] Ujunwa FA , Ikefuna AN , Nwokocha AR et al. (2013) Hypertension and prehypertension among adolescents in secondary schools in Enugu, South East Nigeria. Ital J Pediatr 39, 70. 24180427 10.1186/1824-7288-39-70 PMC4228429

[14] Olumakaiye M , Atinmo T & Olubayo-Fatiregun MA (2010) Food consumption patterns of Nigerian adolescents and effect on body weight. J Nutr Educ Behav 42, 144–151. 20083439 10.1016/j.jneb.2008.12.004

[15] Onabanjo OO & Balogun OL (2014) Anthropometric and iron status of adolescents from selected secondary schools in Ogun State, Nigeria. ICAN Infant Child Adolesc Nutr 6, 109–118.

[16] Wrottesley SV , Mates E , Brennan E et al. (2023) Nutritional status of school-age children and adolescents in low- and middle-income countries across seven global regions: a synthesis of scoping reviews. Public Health Nutr 26, 63–95. 35156607 10.1017/S1368980022000350 PMC11077463

[17] Sosanya M , Freeland-Graves J , Gbemileke A et al. (2021).Why are you so thin? Exploring individual and household level factors of adolescent girls’ food consumption in rural Bauchi, Northern Nigeria. Curr Dev Nutr 5, 182.

[18] Shapu RC , Ismail S , Ahmad N et al. (2020).Knowledge, attitude, and practice of adolescent girls towards reducing malnutrition in Maiduguri metropolitan council, Borno State, Nigeria: cross-sectional study. Nutrients 12, 1681. 32512907 10.3390/nu12061681 PMC7353014

[19] Patton GC , Neufeld LM , Dogra S et al. (2022) Nourishing our future: the Lancet Series on adolescent nutrition. Lancet 399, 123–125. 34856189 10.1016/S0140-6736(21)02140-1

[20] High Level Panel of Experts on Food Security and Nutrition (2017) *Nutrition and Food Systems. A Report by the High Level Panel of Experts on Food Security and Nutrition of the Committee on World Food Security*. Rome: High Level Panel of Experts on Food Security and Nutrition.

[21] Famine Early Warning Systems Network (2018) Nigeria Livelihood Zone Map and Descriptions. Washington, DC: Famine Early Warning Systems Network.

[22] USAID Advancing Nutrition (2024) Guidelines for Market-Based Food Environment Assessments. Instruction Manual. Arlington, VA: USAID Advancing Nutrition.

[23] Famine Early Warning Systems Network Nigeria (2023) https://fews.net/west-africa/nigeria (accessed 13 July 2023).

[24] Hennink M & Kaiser BN (2022) Sample sizes for saturation in qualitative research: a systematic review of empirical tests. Soc Sci Med 292, 114523. 34785096 10.1016/j.socscimed.2021.114523

[25] Carlsen B & Glenton C (2011) What about N? A methodological study of sample-size reporting in focus group studies. BMC Med Res Methodol 11, 1–10. 21396104 10.1186/1471-2288-11-26 PMC3061958

[26] Guest G , Namey EE & Mitchel ML (2013) Collecting Qualitative Data: A Field Manual for Applied Research. Thousand Oaks, CA: SAGE Publications.

[27] USAID Advancing Nutrition (2021) Conducting Formative Research on Adolescent Nutrition: Key Considerations. Arlington, VA: USAID Advancing Nutrition.

[28] Global Diet Quality Project (2022) Nigeria Diet Quality Profile. https://www.dietquality.org/countries/nga (accessed 13 July 2023).

[29] Global Diet Quality Project (2023) Diet Quality Questionnaire Tools. https://www.dietquality.org/tools (accessed 13 July 2023).

[30] ATLAS.ti (2021) Scientific Software Development GmbH (ATLAS.ti 21 Windows). https://atlasti.com (accessed 13 July 2023).

[31] R Core Team (2023) P: A Language and Environment for Statistical Computing. https://www.R-project.org (accessed 13 July 2023).

[32] Okolosi JE (2020) Dietary Pattern, Nutritional Status, and Blood Pressure Level of in-School Adolescents in Edo State, Nigeria. MPH dissertation at the University of Ibadan.

[33] Famine Early Warning Systems Network (2023) Restricted Access to Cash, Anticipated Election Violence, and Persisting Conflict Drive High Food Needs. https://fews.net/west-africa/nigeria/food-security-outlook/february-2023#seasonal-calendar-for-a-typical-year (accessed 13 July 2023).

[34] Garth H (2022) Food, taste, and the body: ingestion and embodiment in Santiago de Cuba. Med Anthropol Q 37, 5–22. 36367138 10.1111/maq.12738

[35] Fleming CAK , de Oliveira JD , Hockey K et al. (2020) Food and Me. How adolescents Experience Nutrition across the World. A Companion Report to The State of the World’s Children 2019. Sydney: Western Sydney University and United Nations Children’s Fund (UNICEF). doi: 10.26183/26f6-ec12.

[36] Neufeld LM , Andrade EB , Ballonoff Suleiman A et al. (2022) Food choice in transition: adolescent autonomy, agency, and the food environment. Lancet 33, 185–197. 10.1016/S0140-6736(21)01687-1 34856191

[37] Boyland E , McGale L , Maden M et al. (2022) Association of food and nonalcoholic beverage marketing with children and adolescents’ eating behaviors and health: a systematic review and meta-analysis. JAMA Pediatr 176, e221037. 35499839 10.1001/jamapediatrics.2022.1037 PMC9062773

[38] World Health Organization (2022) Food Marketing Exposure and Power and their Associations with Food-Related Attitudes, Beliefs and Behaviours: A Narrative Review. Geneva: World Health Organization.

[39] Mintz SW (1996) Tasting Food, Tasting Freedom: Excursions into Eating, Culture, and the Past. Boston: Beacon Press.

[40] Bourdieu P (1984) Distinction: A Social Critique of the Judgement of Taste. Cambridge, MA: Harvard University Press.

[41] Wilk R (2012) Loving people, hating what they eat: marginal foods and social boundaries. In Reimagining Marginalized Foods: Global Processes, Local Places, pp. 15–33 [ E Finnis , editor]. Tucson: The University of Arizona Press.

[42] Neely E , Walton M & Stephens C (2014) Young people’s food practices and social relationships. A thematic synthesis. Appetite 82, 50–60. 25017130 10.1016/j.appet.2014.07.005

[43] Mercy Corps (2013) Adolescent Girls in Northern Nigeria: Financial Inclusion and Entrepreneurship Opportunities Profile. Portland, OP: Mercy Corps.

[44] Hargreaves D , Mates E , Menon P et al. (2022) Strategies and interventions for healthy adolescent growth, nutrition, and development. Lancet 399, 198–210. 34856192 10.1016/S0140-6736(21)01593-2

[45] Blum LS , Khan R , Sultana M et al. (2019) Using a gender lens to understand eating behaviours of adolescent females living in low-income households in Bangladesh. *Matern Child Nutr* 15, e12841. 10.1111/mcn.12841 PMC6852560 31083774

[46] Varela P , Rosso SD , Moura AF et al. (2023) Bringing down barriers to children’s healthy eating: a critical review of opportunities, within a complex food system. Nutr Res Rev. Published online: 25 September 2023. doi: 10.1017/S0954422423000203. 37746804

