# Supplementary material for: Food choice, embodied knowledge and circumscribed agency: factors influencing adolescent girls’ and boys’ dietary practices in three states in northern Nigeria
Source: Public Health Nutr. 2024 Oct 10;27(1):e208. doi: 10.1017/S1368980024001460 (PMC11604317; doi:10.1017/S1368980024001460)
Supplement: Conrad et al. supplementary material [file S1368980024001460sup001.docx]

# Supplementary Materials

## Supplementary Table 1. Distribution of different food groups consumed by adolescent boys and girls

|  | **All** | **Girls (10–14 years)** | **Non-pregnant girls (15–19 years)** | **Pregnant girls (15–19 years)** | **Boys (10–14 years)** | **Boys (15–19 years)** | **Urban** | **Rural** |
| --- | --- | --- | --- | --- | --- | --- | --- | --- |
|  | n (%) | | | | | | | |
| n | 180 | 36 | 36 | 36 | 36 | 36 | 90 | 90 |
| **Food groups** |  |  |  |  |  |  |  |  |
| Grains | 179 (99%) | 36 (100%) | 36 (100%) | 36 (100%) | 35 (97%) | 36 (100%) | 90 (100%) | 89 (99%) |
| Pulses | 133 (74%) | 27 (75%) | 28 (78%) | 23 (64%) | 26 (72%) | 29 (81%) | 66 (73%) | 67 (74%) |
| Nuts and seeds | 88 (49%) | 16 (44%) | 16 (44%) | 15 (42%) | 21 (58%) | 20 (56%) | 44 (49%) | 44 (49%) |
| Dairy | 106 (59%) | 22 (61%) | 21 (58%) | 20 (56%) | 21 (58%) | 22 (61%) | 56 (62%) | 50 (56%) |
| Meat, poultry, fish | 117 (65%) | 18 (50%) | 30 (83%) | 23 (64%) | 22 (61%) | 24 (67%) | 56 (62%) | 61 (68%) |
| Eggs | 43 (24%) | 6 (17%) | 4 (11%) | 11 (31%) | 10 (28%) | 12 (33%) | 21 (23%) | 22 (24%) |
| Dark green leafy vegetables | 117 (65%) | 25 (69%) | 19 (53%) | 28 (78%) | 22 (61%) | 23 (64%) | 54 (60%) | 63 (70%) |
| Other vitamin A-rich fruits and vegetables | 118 (66%) | 23 (64%) | 18 (50%) | 24 (67%) | 24 (67%) | 29 (81%) | 64 (71%) | 54 (60%) |
| Other vegetables | 144 (80%) | 32 (89%) | 25 (69%) | 30 (83%) | 26 (72%) | 31 (86%) | 68 (76%) | 76 (84%) |
| Other fruit | 113 (63%) | 22 (61%) | 15 (42%) | 23 (64%) | 23 (64%) | 30 (83%) | 58 (64%) | 55 (61%) |
| **All five recommended food groups** | 100 (56%) | 19 (53%) | 17 (47%) | 19 (53%) | 21 (58%) | 24 (67%) | 50 (56%) | 50 (56%) |
| **Food groups to consume** |  |  |  |  |  |  |  |  |
| Whole grains | 86 (48%) | 14 (39%) | 18 (50%) | 20 (56%) | 18 (50%) | 16 (44%) | 42 (47%) | 44 (49%) |
| Pulses | 133 (74%) | 27 (75%) | 28 (78%) | 23 (64%) | 26 (72%) | 29 (81%) | 66 (73%) | 67 (74%) |
| Nuts and seeds | 88 (49%) | 16 (44%) | 16 (44%) | 15 (42%) | 21 (58%) | 20 (56%) | 44 (49%) | 44 (49%) |
| Vitamin A-rich orange vegetables | 83 (46%) | 15 (42%) | 11 (31%) | 16 (44%) | 18 (50%) | 23 (64%) | 35 (39%) | 48 (53%) |
| Dark green leafy vegetables | 117 (65%) | 25 (69%) | 19 (53%) | 28 (78%) | 22 (61%) | 23 (64%) | 54 (60%) | 63 (70%) |
| Other vegetables | 144 (80%) | 32 (89%) | 25 (69%) | 30 (83%) | 26 (72%) | 31 (86%) | 68 (76%) | 76 (84%) |
| Vitamin A-rich fruits | 89 (49%) | 18 (50%) | 14 (39%) | 17 (47%) | 20 (56%) | 20 (56%) | 51 (56%) | 38 (42%) |
| Citrus fruits | 55 (31%) | 11 (31%) | 10 (28%) | 10 (28%) | 11 (31%) | 13 (36%) | 19 (21%) | 36 (40%) |
| Other fruits | 101 (56%) | 19 (53%) | 13 (36%) | 20 (56%) | 22 (61%) | 27 (75%) | 53 (59%) | 48 (53%) |
| **Food groups to limit** |  |  |  |  |  |  |  |  |
| Soft drinks | 54 (30%) | 14 (39%) | 10 (28%) | 10 (28%) | 8 (22%) | 12 (33%) | 27 (30%) | 27 (30%) |
| Baked/grain-based sweets | 93 (52%) | 21 (58%) | 15 (42%) | 14 (39%) | 23 (64%) | 20 (56%) | 52 (58%) | 41 (46%) |
| Other sweets | 66 (37%) | 14 (39%) | 11 (31%) | 10 (28%) | 17 (47%) | 14 (39%) | 32 (36%) | 34 (38%) |
| Processed meats | 51 (28%) | 7 (19%) | 13 (36%) | 9 (25%) | 13 (36%) | 9 (25%) | 23 (26%) | 28 (31%) |
| Unprocessed red meat | 55 (31%) | 8 (22%) | 12 (33%) | 13 (36%) | 8 (22%) | 14 (39%) | 32 (36%) | 23 (26%) |
| Deep fried food | 75 (42%) | 16 (44%) | 12 (33%) | 13 (36%) | 17 (47%) | 17 (47%) | 40 (44%) | 35 (39%) |
| Fast food and instant noodles | 45 (25%) | 11 (31%) | 5 (14%) | 9 (25%) | 9 (25%) | 11 (31%) | 24 (27%) | 20 (22%) |
| Packaged ultra-processed salty snacks | 37 (21%) | 6 (17%) | 7 (19%) | 4 (11%) | 8 (22%) | 12 (33%) | 13 (14%) | 24 (27%) |

## Supplementary Table 2. Meals and snacks consumed by adolescent girls and boys

|  | **Gender** | | | | | **Residence** | |
| --- | --- | --- | --- | --- | --- | --- | --- |
|  | **Girls**  **(10–14 years)** | **Girls**  **(15–19 years)** | **Pregnant Girls**  **(15–19 years)** | **Boys**  **(10–14 years)** | **Boys**  **(15–19 years)** | **Urban** | **Rural** |
| **Meal (morning)** | | | | | | | |
| Bean porridge | 0 | 0 | 1 | 0 | 0 | 1 | 0 |
| Bread | 0 | 0 | 4 | 0 | 4 | 4 | 4 |
| Instant noodles (Indomie) | 0 | 0 | 1 | 0 | 0 | 0 | 1 |
| Jollof rice | 1 | 1 | 0 | 0 | 2 | 4 | 0 |
| Millet (bulla) | 1 | 3 | 1 | 0 | 0 | 0 | 5 |
| Millet cake (danwake), oil, pepper | 1 | 0 | 0 | 0 | 0 | 1 | 0 |
| Pap with beans cake or bread | 13 | 9 | 4 | 15 | 5 | 21 | 25 |
| Pasta (spaghetti) | 1 | 0 | 4 | 0 | 2 | 7 | 0 |
| Rice (and beans), stew | 8 | 5 | 6 | 9 | 13 | 20 | 21 |
| Rice and yam | 1 | 0 | 0 | 0 | 0 | 1 | 0 |
| Rice ball and soup | 0 | 1 | 3 | 1 | 2 | 1 | 6 |
| Rice cake (masa) | 0 | 0 | 0 | 0 | 5 | 2 | 3 |
| Rice with oil, pepper | 0 | 0 | 0 | 0 | 1 | 1 | 0 |
| Rice, moimoi, with stew | 0 | 0 | 0 | 1 | 0 | 1 | 0 |
| Sorghum drink (Fura) | 0 | 1 | 1 | 3 | 4 | 0 | 9 |
| Swallow (rice, maize, sorghum, corn dough ball) | 8 | 11 | 14 | 9 | 5 | 5 | 42 |
| Sweet potato porridge | 0 | 0 | 1 | 0 | 0 | 1 | 0 |
| Tea | 2 | 0 | 0 | 0 | 0 | 2 | 0 |
| Tea and bread | 0 | 1 | 1 | 0 | 4 | 6 | 0 |
| **Snack (morning)** | | | | | | | |
| Beans cake (kosai) | 2 | 5 | 1 | 3 | 0 | 9 | 2 |
| Biscuit (with or without yogurt) | 0 | 0 | 0 | 4 | 0 | 4 | 0 |
| Bread | 1 | 0 | 0 | 0 | 0 | 1 | 0 |
| Cucumber | 0 | 0 | 0 | 1 | 2 | 0 | 3 |
| Fish | 1 | 0 | 0 | 0 | 0 | 1 | 0 |
| Fruits (orange, mango, banana) | 0 | 2 | 3 | 1 | 3 | 5 | 4 |
| Rice cake (masa) | 0 | 0 | 0 | 0 | 4 | 2 | 2 |
| Soya cake (awara/tofu) | 3 | 4 | 3 | 2 | 2 | 11 | 3 |
| Sugar cane | 0 | 1 | 0 | 6 | 4 | 4 | 7 |
| Sweet/chin-chin | 3 | 2 | 1 | 0 | 1 | 6 | 1 |
| Tea | 0 | 0 | 0 | 1 | 0 | 1 | 0 |
| Tigernut (aya) | 0 | 1 | 0 | 0 | 3 | 2 | 2 |
| **Meal (afternoon)** | | | | | | | |
| Bean ball (alele), stew | 0 | 1 | 0 | 0 | 0 | 1 | 0 |
| Bean cake (kosai) | 0 | 0 | 0 | 1 | 3 | 1 | 3 |
| Gari | 2 | 3 | 1 | 4 | 1 | 2 | 9 |
| Groundnut porridge | 1 | 0 | 0 | 0 | 0 | 1 | 0 |
| Instant noodles (Indomie) | 0 | 0 | 0 | 0 | 1 | 1 | 0 |
| Jollof rice | 3 | 2 | 1 | 1 | 0 | 4 | 3 |
| Pasta (spaghetti) | 3 | 5 | 2 | 1 | 2 | 7 | 6 |
| Rice, beans, oil, and pepper | 4 | 5 | 9 | 7 | 3 | 17 | 11 |
| Rice, stew | 4 | 3 | 11 | 7 | 10 | 28 | 7 |
| Soup (gote tsaki) | 0 | 0 | 0 | 2 | 0 | 0 | 2 |
| Swallow (rice, maize, millet, and guinea corn dough ball) | 8 | 3 | 7 | 8 | 16 | 8 | 34 |
| Yam porridge with water | 1 | 0 | 3 | 0 | 0 | 4 | 0 |
| **Snack (afternoon)** | | | | | | | |
| Beans (alala) | 1 | 1 | 2 | 1 | 1 | 2 | 4 |
| Bean cakes (kosai) | 0 | 2 | 1 | 0 | 6 | 7 | 2 |
| Biscuit (with or without yogurt) | 0 | 0 | 0 | 4 | 1 | 5 | 0 |
| Bread | 0 | 0 | 0 | 1 | 0 | 1 | 0 |
| Cucumber | 0 | 0 | 0 | 1 | 4 | 1 | 4 |
| Egg | 1 | 0 | 0 | 0 | 0 | 1 | 0 |
| Fish | 1 | 0 | 0 | 0 | 0 | 1 | 0 |
| Flour cake (yar lallaba) | 1 | 2 | 0 | 0 | 0 | 0 | 3 |
| Fruits (orange, mango, banana) | 6 | 4 | 3 | 3 | 6 | 14 | 8 |
| Garden eggs (type of eggplant) | 1 | 0 | 0 | 1 | 1 | 0 | 1 |
| Groundnut cake (karago) | 0 | 1 | 1 | 0 | 0 | 1 | 1 |
| Rice cake (masa) | 0 | 2 | 0 | 2 | 7 | 9 | 2 |
| Soya cake (awara) | 8 | 11 | 5 | 4 | 4 | 9 | 23 |
| Sugar cane | 0 | 1 | 0 | 5 | 3 | 1 | 8 |
| Sweets | 0 | 0 | 4 | 2 | 1 | 2 | 5 |
| Tea | 1 | 0 | 0 | 0 | 0 | 1 | 0 |
| Tigernut (aya) | 0 | 1 | 0 | 0 | 0 | 1 | 0 |
| Yam | 1 | 0 | 0 | 1 | 1 | 3 | 0 |
| **Meal (evening)** | | | | | | | |
| Bean balls (alele), stew | 0 | 0 | 0 | 0 | 0 | 0 | 0 |
| Bean cake (kosai) | 0 | 0 | 2 | 1 | 2 | 4 | 1 |
| Gari | 1 | 1 | 2 | 3 | 3 | 5 | 5 |
| Groundnut porridge | 0 | 0 | 0 | 0 | 0 | 0 | 0 |
| Instant noodles (Indomie) | 0 | 0 | 1 | 1 | 5 | 6 | 1 |
| Jollof rice and water | 0 | 0 | 0 | 0 | 1 | 1 | 0 |
| Pasta (spaghetti) | 0 | 1 | 3 | 0 | 0 | 3 | 1 |
| Rice cake (masa) | 0 | 0 | 0 | 0 | 2 | 2 | 0 |
| Rice, beans, oil, and pepper | 5 | 0 | 0 | 1 | 1 | 4 | 3 |
| Sorghum drink (Fura) | 1 | 0 | 1 | 3 | 3 | 2 | 6 |
| Soup (gote tsaki) | 0 | 0 | 0 | 0 | 0 | 0 | 0 |
| Soya cake (awara) | 0 | 0 | 1 | 0 | 0 | 0 | 1 |
| Swallow (rice, maize, millet, and guinea corn dough ball) | 25 | 33 | 36 | 21 | 14 | 55 | 74 |
| Yam porridge with water | 0 | 0 | 0 | 0 | 0 | 0 | 0 |
| **Snack (evening)** | | | | | | | |
| Bean cake (kosai) | 1 | 1 | 4 | 4 | 8 | 15 | 3 |
| Biscuit (and yogurt) | 2 | 0 | 0 | 4 | 1 | 5 | 2 |
| Bread | 1 | 0 | 0 | 1 | 4 | 6 | 0 |
| Couscous | 0 | 0 | 0 | 0 | 1 | 1 | 0 |
| Egg | 0 | 0 | 3 | 0 | 3 | 4 | 0 |
| Fried (chicken, meat, fish) | 0 | 3 | 1 | 1 | 1 | 4 | 2 |
| Fruits (orange, mango, banana, watermelon) | 5 | 5 | 6 | 2 | 4 | 7 | 15 |
| Garden eggs (type of eggplant) | 0 | 0 | 0 | 0 | 1 | 0 | 1 |
| Ginger | 0 | 0 | 0 | 0 | 1 | 1 | 0 |
| Grasshoppers | 0 | 0 | 2 | 0 | 0 | 2 | 0 |
| Groundnut cake (karago) | 0 | 0 | 0 | 1 | 0 | 1 | 0 |
| Instant noodles (Indomie) | 0 | 0 | 0 | 0 | 1 | 1 | 0 |
| Maltina | 0 | 0 | 1 | 0 | 0 | 1 | 0 |
| Millet (drinks) | 0 | 0 | 1 | 0 | 0 | 1 | 0 |
| Potatoes | 0 | 1 | 2 | 0 | 2 | 3 | 2 |
| Rice cake (masa) | 0 | 0 | 0 | 0 | 1 | 1 | 0 |
| Soya cake (awara) | 2 | 5 | 3 | 2 | 4 | 6 | 10 |
| Sugarcane | 0 | 1 | 0 | 2 | 4 | 2 | 5 |
| Yam and egg | 0 | 0 | 3 | 0 | 0 | 3 | 0 |

## Daily schedule

**Supplementary Table 3. Sample daily schedule of adolescent girls and boys**

| **Time of Day** | **Girls** | **Boys** |
| --- | --- | --- |
| Morning | - Pray - Personal hygiene - Cook and eat first meal of the day - House chores - Hawk or trade - Attend Islamic or conventional school (typically if unmarried) | - Pray - Personal hygiene - Farm or rear cattle - Eat first meal of the day - Attend Islamic or conventional school |
| Afternoon | - Cook and eat second meal of the day - House chores (if any) - Pray - Attend Islamic or conventional school (if unmarried) - Hawk, trade, or business activities - Rest or sleep - “Gist” or chat with friends or family (typically if 15–19 years) | - Pray - Eat second meal of the day - Attend Islamic or conventional school - Personal hygiene - Rest or sleep |
| Evening | - Cook and eat third meal of the day - Care for siblings (or children) - Personal hygiene - Pray - Attend Islamic school - House chores and errands - “Gist” or chat with friends or family (typically if 15–19 years) | - Eat third meal of the day - Pray - Attend Islamic school - Engage in business - Personal hygiene - “Gist”, visit, or chat on the phone with friends (typically if 15–19 years) |
